# Ion Channels as Therapeutic Targets for Viral Infections: Further Discoveries and Future Perspectives

**DOI:** 10.3390/v12080844

**Published:** 2020-08-03

**Authors:** Frank W. Charlton, Hayley M. Pearson, Samantha Hover, Jon D. Lippiat, Juan Fontana, John N. Barr, Jamel Mankouri

**Affiliations:** 1 School of Molecular and Cellular Biology, University of Leeds, Leeds LS2 9JT, UK; ed12fwc@leeds.ac.uk (F.W.C.); ed11hmp@leeds.ac.uk (H.M.P.); s.e.hover@leeds.ac.uk (S.H.); j.fontana@leeds.ac.uk (J.F.); j.n.barr@leeds.ac.uk (J.N.B.); 2 School of Biomedical Sciences, University of Leeds, Leeds LS2 9JT, UK; j.d.lippiat@leeds.ac.uk

**Keywords:** virus, ion channel, virus–host interactions, channelopathies, antivirals

## Abstract

Ion channels play key roles in almost all facets of cellular physiology and have emerged as key host cell factors for a multitude of viral infections. A catalogue of ion channel-blocking drugs have been shown to possess antiviral activity, some of which are in widespread human usage for ion channel-related diseases, highlighting new potential for drug repurposing. The emergence of ion channel–virus interactions has also revealed the intriguing possibility that channelopathies may explain some commonly observed virus induced pathologies. This field is rapidly evolving and an up-to-date summary of new discoveries can inform future perspectives. We herein discuss the role of ion channels during viral lifecycles, describe the recently identified ion channel drugs that can inhibit viral infections, and highlight the potential contribution of ion channels to virus-mediated disease.

## 1. Introduction

The human “channelome” contains over 300 known channels [1] that selectively and rapidly transport ions across biological membranes in response to specific stimuli. Ion channels are present on the plasma membranes and organelles of all cells, where they regulate organelle ion homeostasis, mitochondrial function, inflammasome activation, action potential firing, membrane potential, cell volume, and autophagy [2,3,4,5]. Given their importance, it follows that their dysfunctions leads to human diseases, termed channelopathies [6]. These include disease states of the nervous [2], musculoskeletal [5], cardiovascular [7], and immune systems [8]. This has motivated research on compounds that can modulate ion channel activity; ~19% of current FDA-approved drugs are ion channel modulators, second only to drugs targeting G-protein coupled receptors [9,10].

Many viruses encode their own ion channels [11,12,13] termed “viroporins,” highlighting the importance of ionic balance during viral infection. This field has spurred intense research and several drugs that target viroporins have emerged (reviewed in [11]). More recent evidence highlights how viruses can regulate and/or depend on the ion channels expressed by host cells, highlighting them as new host targets for therapeutic intervention (reviewed by Hover et al., 2017) [14]. Given recent and important advances in this field, we herein provide an up-to-date review of the virus–ion channel literature and discuss the future prospects of ion channel drugs as anti-viral agents. Firstly, we highlight recent evidence that suggests that viruses have adapted to take advantage of endolysosomal ionic balance as a cue for viral entry. We then discuss how intracellular ion channels contribute to the efficiency of viral replication. Finally, we describe how viral infections may result in ion channel dysfunction and lead to virus-induced channelopathies in infected individuals.

## 2. Ionic Balance in the Endosomal System

Most enveloped viruses enter cells by endocytosis [15]. The endolysosomal system is a dynamic series of intracellular membranous compartments that facilitate the uptake, degradation, and recycling of cellular cargoes and membrane proteins [16]. As endosomes mature, they undergo morphological and biological changes (reviewed in Scott et al., 2011) [17]. During early endosomal maturation, the intraluminal pH becomes more acidic, through the action of vacuolar-ATPases (v-ATPases). v-ATPases acidify intracellular vesicles through ATP-driven proton transport into the intraluminal space [18]. To mitigate the large positive charge within endosomes, chloride (Cl^−^) flows inwards through endosomal anion channels, accompanied by cation efflux via Na^+^K^+^/H^+^ exchangers [19,20,21,22,23,24]. Na^+^/K^+^-ATPases are present on early endosomes where they transport Na^+^ into the lumen to limit proton influx, and in turn, acidification [25]. Ca^2+^ plays a number of regulatory roles throughout the endosomal network, including the regulation of fusion and fission events, lipid trafficking, and lysosomal activity [26,27]. The role of K^+^ influx into endosomes is to-date uncharacterised, but an increase in luminal K^+^ occurs as endosomes mature [28]. Whilst the role of low pH in viral entry is well documented [29], the role of other endolysosomal ions is only beginning to be appreciated (Table 1).

## 3. Ion Channels Involved in Viral Entry

### 3.1. Ca^2+^ Channels and Viral Entry

The involvement of Ca^2+^ channels during viral entry is now well-documented [26]. Fujioka et al. showed that influenza virus (IAV) hemagglutinin (HA) triggers intracellular [Ca^2+^] oscillations that are required for viral infection [30]. The initial modulation of Ca^2+^ by IAV was demonstrated using Förster resonance energy transfer (FRET)-based imaging of the Ca^2+^ sensor Yellow Cameleon (YC3.60). These oscillations in Ca^2+^ were mediated by a specific voltage-gated Ca^2+^ channel (Ca_V_1.2) identified through siRNA silencing approaches. Assays subsequently revealed that IAV directly binds to Ca_V_1.2 via the interaction of HA and a sialylated site on Ca_V_1.2. Accordingly, IAV entry could be inhibited by diltiazem, a clinically available Ca^2+^ blocker, highlighting the potential of these compounds for drug repurposing. 

**Figure 1:**
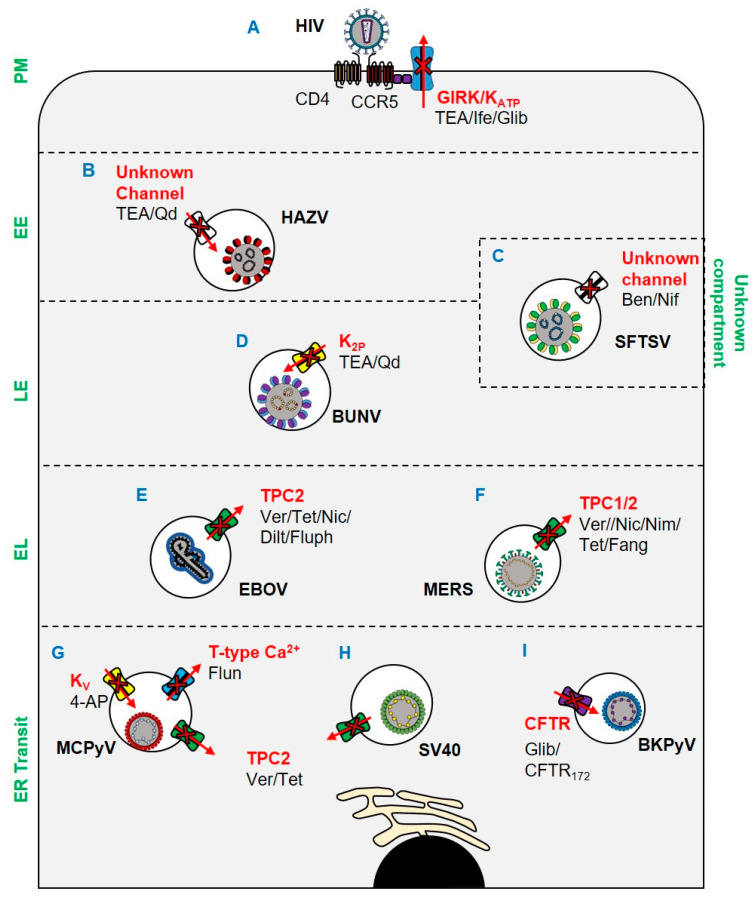
Ion channels implicated in viral entry since 2017. (**A**) Ifenprodil, glibenclamide, and TEA inhibit HIV through blockade of GIRK channels and K_ATP_ channels. (**B**) TEA and quinidine inhibit HAZV escape from EEs via blockade of an unknown channel. (**C**) Endosomal escape of SFTSV is inhibited by benidipine hydrochloride and nifedipine. (**D**) BUNV escape from late endosomes is inhibited by K_2P_ blockade. (**E**) EBOV escape from lysosomes is TPC2-dependent and can be blocked by verapamil, tetrandrine, nicardipine, diltiazem, and fluphenazine. (**F**) MERS escape from endolysosomes is prevented by tetrandrine, fangchinoline, verapamil, nimodipine, and nicardipine blockade of TPCs. (**G**) MCPyV and (**H**) SV40 ER translocation is TPC2 mediated and can be inhibited by verapamil and tetrandrine. ER translocation of MCPyV is also susceptible to blockade of K_V_ and T-type VGCCs by 4-AP and flunarizine respectively. (**I**) ER trafficking of BKPyV is CFTR dependent and susceptible to blockade by CFTR172 and glibenclamide. Key: PM: plasma membrane; EE: early endosome; LE: late endosome; EL: endolysosome. Ver: verapamil; Tet: tetrandrine; Nic: nicardipine; Dilt: diltiazem; Fluph: fluphenazine; Fang: fangchinoline; Nim: nimodipine; Nif: nifedipine; TEA: tetraethylammonium; Qd: quinidine; Ife: ifenprodil; Glib: glibenclamide; 4-AP: 4-aminopyridine.

Ebola virus (EBOV) also requires Ca^2+^ channels for its entry into host cells. EBOV enters cells through endolysosomes positive for both Niemann–Pick C1 (NPC1) and two-pore Ca^2+^ channel 2 (TPC2) [32]. To further characterise this pathway, Penny et al. expanded the pharmacology of TPCs using a virtual screen of ~1500 FDA-approved drugs. All identified TPC modulators were cross-referenced with two recent anti-EBOV screens, with four dopamine receptor antagonists and five oestrogen receptor modulators identified. As such, it was reasoned that these drugs exert their inhibitory effects on EBOV through the blockade of TPCs (Figure 1E), subsequently confirmed through EBOV virus-like particle (VLP) assays [33]. Das et al. further characterised the role of Ca^2+^ in EBOV entry using single-molecule FRET (smFRET)-imaging. It was shown that Ca^2+^ and pH synergistically induce a conformational change in the EBOV glycoprotein GP2 (a key mediator of receptor binding and viral entry) to form a reversible intermediate state primed for NPC1 binding. NPC1 binding then further promotes the conformational transition into a fusion-ready “primed” state of invading EBOV virions [43].

Of importance to the current pandemic, it has been shown that Middle East respiratory syndrome coronavirus (MERS) [44] is dependent on TPCs to escape endosomes [34]. As an enveloped virus, MERS must fuse its envelope with host membranes to enter cells. Following receptor attachment, MERS particles can fuse at either the cell surface or intracellularly in the endosomal network. Fusion is mediated by the proteolytic cleavage of the viral spike (S) protein at its S1/S2 site. At the surface of the cell, this proteolytic event is facilitated by TMPRSS2, which in turn precludes exposure of the fusion loop and coalescence of host and viral membranes [45]. Alternatively, fusion can occur in late endosomes following translocation through the endocytic network and proteolytic processing by proprotein convertases, including furin, in a process regulated by Ca^2+^ [46,47]. In studies by Gunaratne et al., genetic silencing of TPC1 and TPC2 prevented the entry of pseudotyped MERS (Figure 1F). The dependence of MERS upon TPCs was further demonstrated through its inhibition by tetrandrine and fangchinoline (TPC inhibitors), which prevented a post-internalisation but pre-fusion entry event. The mechanism through which TPC blockade inhibited MERS was multi-faceted: TPC1 and TPC2 silencing impaired furin activity, whilst pharmacological and genetic inhibition of TPC1 impaired endosomal motility. Of note, the related SARS-CoV-2, the causative agent of COVID-19 [48,49], was similarly inhibited by TPC blockade. Specifically, treatment of cells with tetrandrine reduced the entry of a lentiviral vector pseudotyped with the SARS-CoV-2 S (spike) [36].

The bunyavirus severe fever with thrombocytopenia syndrome virus (SFTSV) is an emerging arbovirus with fatality rates of 12–50% and the potential to cause future pandemics [35]. Using a library of FDA-approved drugs, the Ca^2+^ channel blockers benidipine hydrochloride and nifedipine were shown to inhibit SFTSV infection (Figure 1C), with in vivo activity confirmed using C57BL/6 and humanised mouse models. Through a retrospective analysis of human SFTSV cases, clinical evidence of the efficacy of nifedipine as an anti-SFTSV therapeutic was also demonstrated. A cohort of patients receiving nifedipine prior to and during hospital admission showed enhanced viral clearance and reduced frequency of neurological syndromes, often associated with fatal outcomes [50,51]. The fatality rate of patients receiving nifedipine was reduced 5-fold compared to untreated patients, which corresponded to abnormal serum Ca^2+^ levels at admission. The viral processes through which SFTSV requires Ca^2+^ channels were subsequently shown to be during virus internalisation and genome replication.

### 3.2. K^+^ Channels and Viral Entry

**Figure 2:**
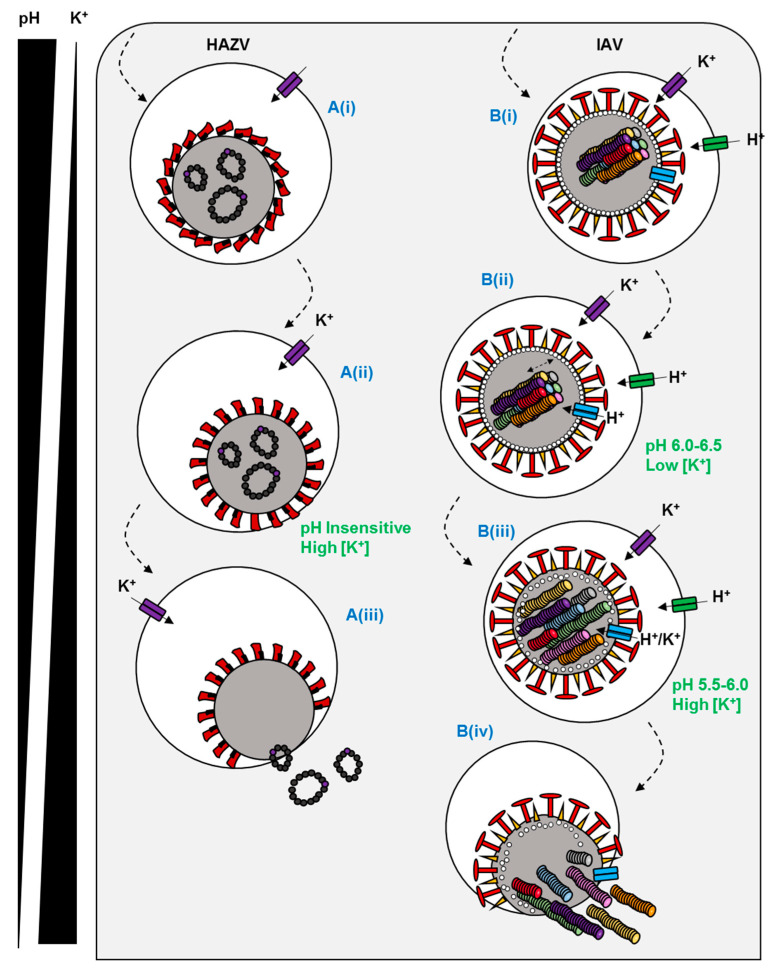
Predicted mechanisms of ion channel dependence for two enveloped viruses. (**A**(**i**)) HAZV is endocytosed by an undefined mechanism. (**A**(**ii**)) Endosomal K^+^ influx triggers a conformational change in the HAZV glycoprotein spikes to a fusion-ready state. (**A**(**iii**)) Host and viral membranes fuse and RNPs are liberated into the cytosol. (**B**(**i**)) IAV is endocytosed via a clathrin-dependent or independent mechanism. (**B**(**ii**)) The virus traffics to late endosomes where the M2 viroporin is activated by acidic pH. (**B**(**iii**)) The influx of K^+^ and H^+^ destabilises matrix-RNP interactions in the core. (**B**(**iv**)) At low pH, a conformational change in HA promotes fusion and RNP release.

The involvement of K^+^ channels in viral entry has been extensively characterised for the model bunyaviruses Bunyamwera orthobunyavirus (BUNV); and Hazara orthononairovirus (HAZV), a model for Crimean Congo haemorrhagic fever virus, which causes severe viral haemorrhagic fever outbreaks, with a case fatality rate of up to 40%. Initial work using known K^+^ channel pharmacology suggested that the blockade of two-pore K^+^ channels (K_2P_) inhibited the early stages of the BUNV lifecycle (Figure 1D) [37]. Subsequent work identified both acidic pH and K^+^ in endosomes as crucial biochemical cues for the endosomal escape of BUNV [28]. Similar studies in HAZV highlighted a dependence on K^+^ channels for infection, and that K^+^ primarily accumulated in cholesterol-rich endosomes (Figure 1B) [28,38,39]. The K^+^ dependence of HAZV involves the glycoprotein spikes; a change in K^+^ concentration triggers conformational changes in the glycoproteins, as revealed through cryo-electron tomography of HAZV virions incubated with K^+^ that “primed” them for insertion into target membranes (Figure 2A). Moreover, it was shown that both BUNV and HAZV could be “primed” in vitro in buffers containing high [K^+^], which expedited entry and subsequent viral gene expression. This phenomenon was analogous to earlier studies for IAV, in which acid bypass in the presence of K^+^ revealed that the exposure of IAV virions to low pH and high [K^+^] weakened interactions between the M1 matrix protein and ribonucleoprotein (RNP) bundles, a pre-requisite for genome release (Figure 2B). The exposure of IAV virions to K^+^ therefore drives viral uncoating and expedites IAV infection [31].

Recent work also highlights a requirement for K^+^ channels during human immunodeficiency virus (HIV) infection. Using pharmacological approaches (Figure 1A) [40] HIV entry could be blocked with ifenprodil and the broad spectrum K^+^ channel blocker tetraethylammonium (TEA). Khan et al. also showed that the pharmacological activation of the endolysosome-resident transient receptor potential mucolipin 1 channel (TRPML1) enhanced the degradation of HIV-Tat (a multi-function viral protein involved in transcription, splicing, capping, and translation), which in turn reduced the transition from viral latency [52]. TRPML1 activates large conductance Ca^2+^-activated potassium (BK) channels in endosomes [53], implying that this channel is required for HIV infection.

The reliance of viruses upon ion channels is not restricted to RNA viruses. It was recently shown that both K^+^ and Ca^2+^ channels are important host factors for polyomavirus infection [41]. Using a panel of ion channel modulators, the entry of Merkel cell polyomavirus (MCPyV), the causative agent of Merkel cell carcinoma (MCC), was shown to be sensitive to 4-aminopyridine (4-AP), a blocker of voltage-gated K^+^ (K_V_) channels (Figure 1G). Moreover, both MCPyV and Simian virus 40 (SV40) (Figure 1H) were sensitive to verapamil, a broad-spectrum Ca^2+^ channel blocker. The identities of the Ca^2+^ channels involved in polyomavirus entry were further explored, revealing a requirement for transient (T-type, low-voltage activated) channel family members in MCPyV infection but not SV40. Tetrandrine, a TPC blocker, restricted both viruses. The role of TPCs was found to be during endoplasmic reticulum (ER) disassembly and/or ER docking for SV40 [54], which may be explained by the recent demonstration that Ca^2+^ ions mediate the stabilization of SV40 capsids and contribute to its disassembly [55].

### 3.3. Cl^−^ Channels and Viral Entry

BK polyomavirus (BKPyV) is a potentially fatal pathogen in patients undergoing solid organ transplantation. Panou et al. demonstrated that the pharmacological and genetic disruption of the cystic fibrosis transmembrane conductance regulator (CFTR) Cl^−^ channel could reduce BKPyV infection in primary kidney cell models (Figure 1I) [42]. Time of addition assays using the CFTR inhibitors CFTR_172_ and glibenclamide, combined with the assessment of exposure of VP2/VP3 minor capsid proteins, indicated a role for CFTR in the trafficking of BKPyV to the ER. Whilst the mechanism of CFTR involvement in BKPyV ER trafficking remains unclear, it is hypothesised that the channel may be important in the acidification and ER docking of BKPyV-containing endosomes. CFTR has been implicated in the fusion of endosomes [56], and co-localises with vacuolar ATPase (vATPase) to provide the counter-charge during endosomal acidification [57].

## 4. Ion Channels in Viral Replication

Once viral genomes are released inside the host cell, replication can commence. Recent evidence suggests that this process is partially controlled by ion concentrations and can therefore be targeted by ion channel drugs.

### 4.1. Ca^2+^ Channels and Viral Replication

Flaviviruses establish replication complexes in modified intracellular membranes, often derived from the ER. The ER stores the majority of intracellular Ca^2+^, and so it is perhaps unsurprising that an array of flaviviruses depend on intracellular Ca^2+^ ion channels for their replication (Table 2). Japanese encephalitis virus (JEV) is an arthropod-borne virus linked to acute encephalitis. Wang et al. performed a screen of FDA-approved drugs to assess in vitro activity against JEV [58]. Within the screen, three of the five most potent inhibitors were blockers of voltage-gated Ca^2+^ channels (VGCCs), including manidipine, cilnidipine, and benidipine hydrochloride. Time of addition assays suggested that these three drugs did not act during viral entry, nor were they virucidal, but specifically inhibited virus replication. The potential of these drugs as broad-acting anti-flavivirus treatments was further assessed and each led to a concentration-dependent inhibition of Dengue virus DENV), West Nile virus, and Zika virus (ZIKV) replication. Yellow fever virus (YFV) was, however, insensitive to manidipine. Interestingly, the in vitro selection of a manidipine-resistant JEV identified a Q130R mutation in the non-structural protein NS4B. Sequence alignments confirmed that the Q130 site was conserved in each of the flaviviruses susceptible to manidipine, but not YFV. The efficacy of manidipine against JEV was further confirmed in in vivo mouse models, with manidipine-treated mice exhibiting significantly higher survival rates compared to untreated mice challenged with JEV.

A role for Ca^2+^ channels during DENV replication was further revealed by Dionicio et al. [59]. DENV was shown to inhibit intracellular Ca^2+^ release from the ER, which in turn activated the store-operated Ca^2+^ (SOCE) pathway. In addition, specialised Ca^2+^ release activated Ca^2+^ channels (CRACs) were identified as a requirement for DENV replication. Small molecule blockers of these channels resulted in a 70% reduction in virus yield. Additionally, yeast two-hybrid screens identified the Ca^2+^-permeable non-selective transient receptor potential vanilloid 4 (TRPV4) in complex with the DEAD-box helicase (DDX3X) as a key regulator of viral mRNA translation for ZIKV [60].

The hepatitis B virus (HBV) X protein is an oncoprotein that regulates cytosolic [Ca^2+^] [61]. Recently, Yao et al. characterised this mechanism, revealing its interaction with ORAI1, a critical component of the SOCE pathway [62]. HBV replication was also dependent on K^+^ in studies by Chakraborty et al. [63]. They reported that K^+^-dependent nucleolytic activity in the presence of HBV RNA mediates the self-cleavage of a 53 nt oligomer with ribozyme activity that is required for viral replication.

### 4.2. Cl^−^ Channels or Other Anions and Vials Replication

Chikungunya virus (CHIKV) is a re-emerging arbovirus associated with long-term complications and high morbidity. Using siRNA silencing of the Cl^−^ intracellular channels (CLIC) 1 and 4, Müller et al. demonstrated a requirement for both channels during the replication of a CHIKV sub-genomic replicon in mammalian and invertebrate cells [65]. The voltage-dependent anion channel 1 (VDAC1) is also implicated in viral replication; it is upregulated by infectious bursal disease virus (IBDV). Han et al. showed that knockdown of VDAC1 inhibited IBDV replication through the reduction of viral polymerase activity, and that the overexpression of VDAC1 promotes polymerase activity. Immunoprecipitation (IP) experiments showed that VDAC1 interacts with IBDV VP1 and VP3, components of RNPs, indicating a role for this channel in RNP formation [64].

## 5. Viruses as Causative Agents of Acquired Channelopathies

Viral pathologies are becoming increasingly linked to the dysregulation of host ion channels (Table 3). This reveals an interesting and new avenue for ion channel drugs, as the pharmacological manipulation of virus-targeted channels may not only impair viral infection at the cellular level, but may circumvent virus-induced channelopathies.

### 5.1. Viral Channelopathies and Ca^2+^ Channels

Recent studies have linked viral infection to neuronal pathologies through the dysregulation of Ca^2+^ signalling. The FDA-approved Alzheimer’s drug memantine protected against neuronal cell death induced by ZIKV infection [66]. Memantine acts upon the N-methyl-d-aspartate receptor (NMDAR), which mediates Ca^2+^ signalling to govern synaptic plasticity [67]. The overstimulation of NMDAR is linked to neurodegeneration, a pathology commonly associated with ZIKV. Upon challenge with memantine, ZIKV replication was unaffected, but antagonism of NMDAR invoked a neuroprotective effect in vivo. Whilst the exact mechanism(s) of ZIKV neuropathologies are unknown, it is predicted that the virus hyper-stimulates NMDAR to upregulate Ca^2+^ signalling to the point of Ca^2+^ overload and postsynaptic neuronal death, a process termed “glutamate excitotoxicity.”

The reactivation of herpes simplex virus 1 (HSV-1) can lead to cranial nerve disorders and severe pain. Zhang et al. revealed that HSV-1 disrupts the expression of T-type Ca^2+^ channels in differentiated sensory-like neurons, as a means to disrupt pain responses [68,69]. Proteomics and transcriptomics showed that HSV-1 decreased the expression of the Ca_V_3.2 T-type Ca^2+^ channel subunit at the protein level, despite increasing Ca_V_3.2 mRNA synthesis. This upregulation of Ca_V_3.2 mRNA synthesis was postulated to be a compensatory response to decreased expression of the channel subunit. The loss of Ca_V_3.2 initially led to reduced pain transmission in infected neurons; however, the release of interleukin-6 (IL-6) in response to viral infection was subsequently shown to restore Ca_V_3.2 current density and pain responses. 

Rotavirus (RV) dysregulates cellular Ca^2+^ homeostasis through the depletion of ER stores. Using genetically-encoded Ca^2+^ indicators in infected cells, cytosolic Ca^2+^ increased in distinct peaks [70,71,72], which was mediated by the non-structural protein NSP4. RV-NSP4 acts as a Ca^2+^-permeable viroporin to release Ca^2+^ from the ER, which in turn activates the ER Ca^2+^ sensor STIM1, subsequently leading to SOCE activation. RV-infected cells then secrete a cleavage product of NSP4 (eNSP4), which elicits an inward inositol triphosphate (IP_3_)-dependent Ca^2+^ signal, which in turn stimulates Cl^−^ release through Ca^2+^-activated Cl^−^ channels (CaCCs) [73]. The efflux of Cl^−^ from cells is a known causative factor of diarrhoea in vivo, thereby identifying NSP4 as the first viral enterotoxin. This multi-faceted control of Ca^2+^ signalling suggests a crucial role for Ca^2+^ channels in the pathophysiology of RV. In this regard, it was shown that the blockade of CaCCs, including TMEM16A, reduces intestinal motility and fluid loss in vivo with no direct effects on the levels of virus infection [74].

### 5.2. Viral Channelopathies Associated with K^+^ Channels

Coxsackie virus B3 (CVB3), amongst other enteroviruses, is associated with cardiomyopathies and sudden cardiac death [76]. KCNQ1 is a K_V_ channel (K_V_7.1) that mediates a delayed, slow rectifying K^+^ current in ventricular tissue to regulate contractility. Kv7.1 trafficking and activity are regulated by the serine/threonine kinase SGK1 [80], which is upregulated by CVB3. As such, KCNQ1 currents are elevated to 125% in CVB infected cells, whilst hERG1 (K_V_11.1) and Ca_V_1.2 activity decrease by 59% and 83% respectively. These results corroborated with localisation studies of each channel and demonstrated that the surface expression of KCNQ1 increased, whilst hERG1 and Ca_V_1.2 expression decreased in infected cells. Inherited mutations in each of these three ion channels are associated with heart rhythm disorders. Importantly, decreased hERG1 expression increases the risk of drug-induced arrhythmias; the additional inhibition or reduced trafficking of this channel in cells targeted by small therapeutic molecules reduces the number of redundant repolarisation currents, in turn depleting the overall repolarisation reserve. Together, these data suggest that CVB3 re-programmes ion channel expression in cardiac tissue, leading to an increased risk of arrhythmia. This highlights these channels as therapeutic targets to prevent the sudden cardiac death that results from CVB3 infection.

### 5.3. Viral Channelopathies Associated with Na^+^ Channels

A number of viruses that primarily infect the airway system have been shown to dysregulate airway epithelial Na^+^ transport. Human respiratory syncytial virus (HRSV) primarily infects airway epithelial cells, and dysregulates epithelial Na^+^ channels (ENaC) to disrupt Na^+^ flux in the airways [81]. ENaCs are a critical mediator of osmotic fluid absorption across airway epithelia, through the selective transport of Na^+^. Electrochemical balance is maintained through apical Cl^−^ channels, which include CFTR. Clinical studies of infants diagnosed with HRSV showed a negative correlation between ENaC mRNA expression and the severity of HRSV bronchiolitis [82]. HRSV has been shown to manipulate airway ion flux through upregulation of channels involved in the cough reflex, namely, transient receptor cation channel, subfamily A, member 1 (TRPA1), and the acid sensing ion channel receptor 3 (ASIC3), a member of the ENaC family of Na^+^ channels [77]. HRSV induced a 30-fold increase in ASIC3 mRNA in normal bronchial epithelial cells, compared to the 3-fold increase observed in cells challenged with measles virus (MeV). Interestingly, UV-inactivated HRSV and MeV maintained their ability to upregulate ASIC3, suggesting these effects were independent of genome replication. The virion-induced upregulation of IL-6 and IL-8 was subsequently shown to inhibit TRP receptor activity, identifying these receptors as potential targets for virus-induced cough symptoms.

ENaC and CFTR channel expression are also modulated by IAV. From single-cell recordings in intact lung epithelial cells, Brand et al. described a reduction in ENaC and CFTR activity upon IAV-infection, through reduced surface expression. The mechanisms governing this downregulation were not characterised, but it is thought that IAV promote ER stress, known to cause deficits in ENaC abundance [83]. The loss of ENaC and CFTR surface expression was accompanied by a reduction in airway surface liquid (ASL), a known contributor to cystic fibrosis. Importantly, treatment with the FDA-approved CFTR corrector lumacaftor could restore IAV-mediated ASL perturbations, highlighting how virus-induced pathologies can be treated by therapies targeting host ion channels [78].

The altered activity of Na^+^ channels was observed in cells latently infected HSV-1 dorsal root ganglion neurones. Upon acute lytic infection, HSV-1 was found to reduce functional voltage-gated sodium channel (VGSC) expression within 24 h and abolish VGSC activity within 3 days, whilst latent HSV-1 infection led to a recovery of these currents and increased Na_V_1.7 channel expression. Furthermore, HSV-1 reactivation from a dormant state decreased VGSC activity. It is known that VGSCs play a role in the transmission of pain signals [75]. Similarly, post-herpetic neuralgia associated with varicella-zoster virus is associated with an increase in Na^+^ current amplitude through the activity of Na_V_1.6 and Na_V_1.7 [84]. Na_V_1.7 dysregulation has also been associated with hereditary pain disorders. Gain-of-function mutations in *SCN9A*, the gene encoding Na_V_1.7, are causative of primary erythromelalgia (PE), a rare neuropathy characterised by recurring pain, warmth, and redness of the extremities. Research into the management of PE identified two novel selective Na_V_1.7 blockers, PF-05089771 and TV-45070, which may hold promise in ameliorating pain symptoms associated with PE [85] and viral-induced neuropathies.

### 5.4. Viral Channelopathies Associated with Cl^−^ Channels

Stakaitytė et al., used a proteomics approach to identify changes in the host channelome in response to the overexpression of MCPyV small tumour antigen (ST). The analysis revealed a role for two CLIC proteins [79], CLIC1 and CLIC4 (4-fold and 5-fold up regulation in cells overexpressing ST vs. control cells, respectively). Pharmacological and genetic inhibition of these channels reduced ST-induced motility and migration, implicating their function in MCPyV, ST-induced metastatic processes [86]. These data were reinforced by the finding that MCPyV-positive MCC tumours showed enhanced CLIC1 and CLIC4 expression (2.5-fold and 3.5-fold increase respectively), implicating their involvement in MCC tumorigenesis. These findings align with earlier evidence that CLIC1 and CLIC4 are involved in the metastatic progression of specific tumour types, through switching of cellular localisation and function to integral transmembrane proteins as active anion channels and signal transducers [87].

## 6. Conclusions and Further Perspectives

It is now clear that host cell ion channels play an important role during viral infection at the cellular level, and as causative factors of disease states in infected tissues. Ion channels have been linked to multiple stages of viral lifecycles, in which viruses are either passively dependent upon or actively modulate channel functionality. Given this knowledge, evidence is emerging that ion channel inhibitors represent a new antiviral strategy. Whilst toxicity profiles for ion channel inhibitors are only available in the context of those used to treat hereditary channelopathies, in vivo evidence is emerging that these drugs can be efficacious against viruses. Moreover, overlapping mechanisms of acquired and hereditary channelopathies may underpin the efficacy of ion channel modulators in treating virally-induced pathophysiologies. The manipulation of host ion homeostasis presents an attractive target for the treatment of many clinically important viruses and their associated pathologies, whilst circumventing the risks of resistance associated with direct-acting antiviral drugs. As such, continued studies of host-virus interactions may guide future antiviral approaches.

## Figures and Tables

**Table 1:** Overview of ion channels implicated in viral entry.

Virus	Ion Channel(s)	Ref.
Influenza A virus (IAV)	Voltage-gated Ca^2+^ channel 1.2 (Ca_V_1.2), Unknown K^+^ channel	Fujioka et al., 2018 [30] Stauffer et al., 2014 [31]
Ebola virus (EBOV)	Two-pore channel 2 (TPC2)	Simmons et al., 2016 [32] Das et al., 2020 [33]
Middle Eastern respiratory syndrome coronavirus (MERS)	Two-pore channels 1 and 2 (TPC1/2)	Gunaratne et al., 2018 [34]
Severe fever with thrombocytopenia syndrome virus (SFTSV)	Unknown channel	Li et al., 2019 [35]
Severe acute respiratory syndrome coronavirus 2 (SARS-CoV-2)	Two-pore channel 2 (TPC2)	Ou et al., 2020 [36]
Bunyamwera orthobunyavirus (BUNV)	Two-pore domain K^+^ (K_2P_)	Hover et al., 2016/18 [28,37]
Hazara orthonairovirus (HAZV)	Unknown K^+^ channel	Punch et al., 2017 [38] Charlton et al., 2019 [39]
Human immunodeficiency virus (HIV)	G-Protein coupled inwardly rectifying K^+^ (GIRK) ATP-sensitive K^+^ K_ATP_	Dubey et al., 2019 [40]
Merkel cell polyomavirus (MCPyV)	Voltage-gated K^+^ (K_V_) T-type Ca^2+^ (Transient, low-voltage activated) Two-pore channel 2 (TPC2)	Dobson et al., 2020 [41]
Simian virus 40 (SV40)	Voltage-gated K^+^ (K_V_) Two-pore channel 2 (TPC2)	Dobson et al., 2020 [41] Panou et al., 2020 [42]
BK polyomavirus (BKPyV)	Cystic fibrosis transmembrane conductance regulator (CFTR)	

**Table 2:** Overview of ion channels implicated in viral replication.

Virus	Ion Channel(s)	Ref.
Japanese encephalitis virus (JEV) West Nile virus (WNV) Dengue virus (DENV)	Voltage-gated calcium channel (VGCCs, Ca_V_)	Wang et al., 2017 [58]
Hepatitis B virus (HBV)	Mitochondrial Ca^2+^ channel	Bouchard et al., 2019 [61]
Dengue virus (DENV)	Two-pore domain K^+^ (K_2P_)	Dionicio et al., 2018 [59]
Infectious bursal disease virus (IBDV)	Voltage-dependent anion channel 1 (VDAC1	Han et al., 2017 [64]
Chikungunya virus (CHIKV)	Cl^−^ intracellular channels (CLIC) 1 and 4	Müller et al., 2019 [65]

**Table 3:** Overview of ion channels implicated in virus-mediated disease.

Virus	Ion Channel(s)	Ref.
Zika virus (ZIKV)	N-Methyl-d-Aspartate receptor (NMDAr)	Costa et al., 2017 [66]
Rotavirus (RV)	Ca^2+^-activated chloride channels (CaCC)	Chang-Graham et al., 2019 [70]
Herpes simplex virus 1 (HSV-1)	T-type Ca^2+^ channels, Voltage-Gated Sodium Channels (VGSCs)	Zhang et al., 2017/19 [68,69] Zhang et al., 2020 [75]
Coxsackie virus B3 (CVB3)	Voltage-gated K^+^ channel (K_V_)	Peischard et al., 2019 [76]
Human respiratory syncytial virus (HRSV)	Epithelial Na^+^ channel (ENaC)	Omar et al., 2017 [77]
Influenza A virus (IAV)	Epithelial Na^+^ channel (ENaC) Cystic fibrosis transmembrane conductance regulator (CFTR)	Brand et al., 2018 [78]
Merkel cell polyomavirus (MCPyV)	Cl^−^ intracellular channels (CLIC) 1 and 4	Stakaitytė et al., 2018 [79]
